# Targeting and Recognition of Toll-Like Receptors by Plant and Pathogen Lectins

**DOI:** 10.3389/fimmu.2017.01820

**Published:** 2017-12-18

**Authors:** Rafael Ricci-Azevedo, Maria-Cristina Roque-Barreira, Nicholas J. Gay

**Affiliations:** ^1^Laboratory of Immunochemistry and Glycobiology, Department of Cell and Molecular Biology and Pathogenic Bioagents, Ribeirão Preto Medical School, University of São Paulo, Ribeirão Preto, Brazil; ^2^Department of Biochemistry, University of Cambridge, Cambridge, United Kingdom

**Keywords:** lectins, carbohydrate recognition domain, N-glycosylation, toll-like receptors, innate immune response

## Abstract

We have reported that some lectins act as agonists of toll-like receptors (TLRs) and have immunomodulatory properties. The plant lectin ArtinM, for example, interacts with N-glycans of TLR2, whereas other lectins of microbial origin interact with TLR2 and TLR4. Expression of the receptors on the surface of antigen-presenting cells exposes N-glycans that may be targeted by lectins of different structures, specificities, and origins. *In vitro*, these interactions trigger cell signaling that leads to NF-κB activation and production of the Th1 polarizing cytokine IL-12. *In vivo*, a same sequence of events follows the administration of an active lectin to mice infected with an intracellular pathogen, conferring resistance to the pathogen. The lectins of the human pathogens *Toxoplasma gondii (TgMIC1 and TgMIC4) and Paracoccidioides brasiliensis* (Paracoccin), by recognition and activation of TLR2 and TLR4, induce cell events and *in vivo* effects comparable to the promoted by the plant lectin ArtinM. In this article, we highlight these two distinct mechanisms for activating antigen-presenting cells. On the one hand, TLRs act as sensors for the presence of conventional pathogen-associated molecular patterns, such as microbial lipids. On the other hand, we showed that TLR-mediated cell activation might be triggered by an alternative way, in which lectins bind to TLRs N-glycans and stimulate cells to increase the expression of pro-inflammatory cytokines. This process may lead to the development of new pharmaceutical tools that promote protective immune responses directed against intracellular pathogens and tumors.

Toll-like receptors (TLRs) are pattern recognition receptors of the innate immune system (1–3). These receptors are Class 1 transmembrane proteins that recognize conserved structures associated with pathogenic microorganisms, collectively designated as pathogen-associated molecular patterns (PAMPs). PAMPs include the bacterial glycolipid LPS, peptidoglycans, and DNA with unmethylated CpG motifs (3). Recognition of PAMPs by TLRs triggers cell signaling and activation, which leads to potent antimicrobial responses and enables the initiation of adaptive Th1 immunity (4, 5). Because it is a desirable response in many circumstances, TLR agonists capable of activating protective mechanisms of the innate immune system are being investigated as possible prophylactic or therapeutic agents to combat infectious or neoplastic diseases (6–9).

It is well established that the architecture of the TLR ectodomains, particularly the solenoidal structure adopted by the leucine-rich repeats (LRR), confers specific recognition of these highly diverse PAMPs. These recognition processes depend on the PAMP structure, the type of TLR involved, the formation of specific heterodimers, and the participation of co-receptors (10, 11). Here, we highlight an alternative mode of TLR signaling activation in which the receptors instead of recognizing PAMPs are activated by sugar binding lectins. Lectins are ubiquitous proteins with reversible and specific carbohydrate recognition activity. By interacting with glycoproteins such as the TLRs on cell surfaces, lectins mediate diverse biological and cellular processes (12).

Toll-like receptor ectodomains are modified by at least four (TLR2) and as many as 9 N-linked glycans (TLR4) (13). These N-glycans play a role not only in biosynthesis and trafficking but also the triggering of TLR signal transduction. TLR N-glycans can be targeted by the carbohydrate recognition domain (CRD) of lectins, directly or indirectly initiating receptor activation (14–16). Lectin binding induces cell signaling and the release of pro-inflammatory cytokines, which can mimic the responses promoted by PAMPs recognition. Thus, these lectins acting through their CRD are potent TLRs agonists.

Pathogen-associated molecular pattern ligands of TLR2 and 4 bind to receptors at the cell surface and induce dimerization of the ectodomains. This causes concerted conformational changes leading to homodimerization of the cytosolic TIR domains, and recruitment of downstream signal transducers, notably MyD88 (17). It is likely that the binding of plant and pathogen lectins to receptor glycans is able to induce an equivalent activation process although this has not yet been experimentally verified. However, it is known that other non-canonical agonists such as nickel ions and cationic lipids stabilize the homodimerization interface that forms between the lateral surfaces of the receptor ectodomains (18) and lectins may have a similar mechanism of action. It is clear that lectins induce production of pro-inflammatory cytokines such as IL-6 and IL-8, but transcriptomic analysis has not been reported. RNASeq experiments currently underway should reveal how the transcriptional program elicited by lectins compares with that of PAMP ligands and whether this constitutes an endogenous alternative TLR pathway.

The first evidence for this alternative TLR activation mechanism was provided by the response of innate immune cells to the plant lectin ArtinM (19). ArtinM is a mannose-binding jacalin-related lectin that has been studied extensively by our research group. This lectin recognizes the N-glycan modifications of both TLR2 and the co-receptor CD14, promoting the assembly of an activation complex on the plasma membrane (15). TLR2 has four N-glycosylation sites in its ectodomain, all of them modified by sugar chains, whose structures are still unknown. The glycan-site 1 is located on the convex surface of the LRR solenoid, exposed to solvent. Sites 2 and 3 are on the concave surface, also in positions that are solvent exposed. By contrast, site 4 is located on the terminal residue of the LRR16, which forms part of the inner surface of the solenoid, in a sterically restricted position. Interestingly, this fourth site is the only one that is conserved in receptors of all studied species (13). TLR2 heterodimerization with TLR1 or TLR6, which occurs as part of the activation process, does not interfere with cellular responses promoted by ArtinM (15). This alternative activation pathway also occurs in response to other plant lectins, as well as to lectins derived from human pathogens. Table 1 summarizes the lectins reported to bind TLRs.

**Table 1:** Plant and pathogens lectins reported to interact with toll-like receptors (TLRs).

Source		Lectin	Carbohydrate specificity	Target	Reference
Plant lectins	*Artocarpus heterophyllus*	ArtinM	Manα1-3[Manα1-6]Man	TLR2/1 and 2/6 +CD14^a^	(15, 19, 20)
	*Canavalia ensiformis*	Concanavalin A	Man/Glc (Man>Glc>GlcNAc)	TLR2/6	(21)
	*Viscum album coloratum*	Korean mistletoe lectin (KML-C)	Terminal Gal and GalNAc residues	TLR4	(22)
	*Phaseolus vulgaris*	Phytohemagglutinin-L (PHA-L)	Galβ1,4GlcNAcβ1,2(Galβ1,4GlcNAcβ1,6Man)	TLR2/6, 4, and 5	(21)
	*Glycine max*	Soybean agglutinin	GalNAc>Gal (terminal Gal and GalNAc residues)	TLR4	(21)
	*Arachis hypogea*	Peanut agglutinin	Gal(Galβ1,3GalNAc) (terminal Gal residue)	TLR4	(21)
	*Triticum vulgaris*	Wheat germ agglutinin	Man/Glc (Man>Glc>GlcNAc)	TLR2/6, 5, 7, and 8	(21)
Pathogens lectins	*Paracoccidioides brasiliensis*	Paracoccin	βGlcNAc14βGlcNAc14GlcNAc	TLR2/1, 2/6, and 4	(14)
	*Toxoplasma gondii*	*T. gondii* microneme 1	NeuAcα-3Galβ-4GlcNAcβ-3Galβ-3GlcNAc (terminal NeuAc residue)	TLR2/1, 2/6, and 4	(23)
	*T. gondii*	*T. gondii* microneme 4	Galβ-3GalNAcβ-4Galβ-4Glc (terminal Gal residue)	TLR2/1, 2/6, and 4	(23)
	*Entamoeba histolytica*	Gal/GalNAc lectin	Terminal Gal/GalNAc residue	TLR2 and 4	(16)

*^a^ArtinM interaction with glycans N-linked to the co-receptor CD14 is required for induction of TLR2 activation (20)*.

Paracoccin (PCN), a lectin derived from *Paracoccidioides brasiliensis*, also exerts immunomodulatory activities due to its interaction with both TLR4 and TLR2 N-glycans. Recent studies have identified the TLR2 N-glycans that are required for triggering responses to the PCN stimulus. The investigation used TLR2 mutants generated by sequentially removing, through site-directed mutagenesis, all four Asn–X–Ser/Thr sequons. The co-transfection of both a mutated-TLR2 and the NF-κB-reporter gene into HEK293 human cells, identified N-glycans that are necessary for mediating responses to the PCN stimulus, in comparison with the response mediated by fully glycosylated TLR2 (14). Interestingly, only the TLR2 N-glycan linked to site 4 of the peptide bone was demonstrated as required for the lectin activity, demonstrating that PCN critically targets the fourth N-glycan to induce TLR2-mediated cell activation. Site 4 is the most conserved and least accessible among all TLR2 sites of N-glycosylation (13). Interestingly, all the TLR2 glycomutants could mediate cell activation in response to a classical agonist such as PAM3CsK4 (Figure 1).

**Figure 1:**
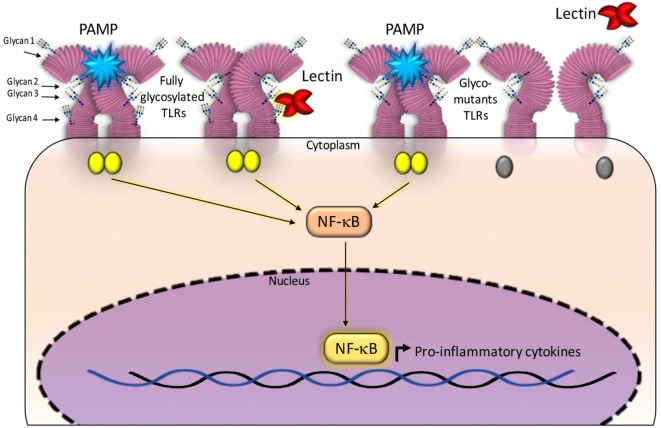
Glycans N-linked to TLR2, expressed on the surface of antigen-presenting cells, are targeted by carbohydrate recognition domain (CRD) of some lectins. The established interactions are followed by intracellular signaling and production of cytokines. Fully glycosylated TLR2 molecules can be targeted by lectins CRDs (shown at the right side of the figure), besides recognizing pathogen-associated molecular patterns (PAMPs) and synthetic ligands. Either interaction induces activation of NF-κB signaling pathway and production of cytokines. Most constructed TLR2 glycomutants (exhibiting isolated or combined elimination of N-glycans) preserve the capacity to recognize PAMPs, whereas some of the glycomutants become selectively unresponsive to a specific lectin stimulus (shown at the left side of this figure). This approach allows defining which N-glycans are critically required to trigger TLR2 activation by a lectin. In this illustration, the TLR2 N-glycans (numbered 1–4 and linked to Asn116, Asn199, Asn416, and Asn442) are not rigorously localized in the TLR2 backbone. For a more realistic representation of N-glycans position on TLR2, see the article authored by Weber et al. (13).

We have also studied a pair of lectins, namely, *T. gondii* microneme 1 (*Tg*MIC1) and *T. gondii* microneme 4 (*Tg*MIC4), originally contained into *Toxoplasma gondii* microneme apical vesicles and early secreted during the process of host cells invasion. These lectins are part of the *Tg*MIC1/*Tg*MIC4/*Tg*MIC6 complex, supported on the parasite surface by *Tg*MIC6, which allows the exposition of *Tg*MIC1 and *Tg*MIC4 CRDs to interact with carbohydrate ligands (glycans with sialic acid and d-galactose on terminal position, respectively) on the host cells. The lectins’ interactions with macrophages and dendritic cells are established by the recognition of TLR2 and TLR4 N-glycans and result in modulation of the immune response toward the Th1 axis. Sardinha-Silva et al. (23) have recently demonstrated the importance of these lectins for the induction of early IL-12 production in *T. gondii* infection. Concerning the discrimination of which TLR2 N-glycans are targeted by *Tg*MIC1 and *Tg*MIC4, those occupying positions 2, 3, and 4, impact the *Tg*MIC1–TLR2 interaction. On the other hand, *Tg*MIC4 requires the TLR2 N-glycans linked to the sites 3 and 4 to interact with the receptor (23).

The characterization of TLRs as targets of recognition by exogenous agents opens new avenues for the design of pharmaceutical tools. Indeed, lectins or biomimetic lectins can be used as TLR agonists to improve immune responses to severe infections, especially in immunosuppressed patients, or as antitumor agents. Lectins might also be used as adjuvants that, associated with conventional treatment, can boost Th1 and Th17 immune responses (24, 25), which are both required to overcome fungal diseases (26). A priority for future research is to define the molecular mechanisms by which pathogen lectins active innate responses.

In conclusion targeting of TLRs by lectins creates a new opportunity to therapeutically manipulate the immune response.

## Author Contributions

Conceived and designed the idea; wrote the text: RR-A, M-CR-B, and NJG. Drew the scheme: RR-A.

## Conflict of Interest Statement

The authors declare that the research was conducted in the absence of any commercial or financial relationships that could be construed as a potential conflict of interest.
